# EDNRA regulates the tumour immune environment and predicts the efficacy and prognosis of cancer immunotherapy

**DOI:** 10.1111/jcmm.70172

**Published:** 2024-11-27

**Authors:** Mengxue Wang, Long Wang, Xunjia Li, Meng Dai, Bo Sheng

**Affiliations:** ^1^ Department of Gastrointestinal Surgery The Second Affiliated Hospital of Chongqing Medical University Chongqing China; ^2^ Department of Breast Cancer Center Chongqing University Cancer Hospital Chongqing China; ^3^ Department of Nephrology Chongqing Traditional Chinese Medicine Hospital Chongqing China; ^4^ Department of oncology Chongqing Traditional Chinese Medicine Hospital Chongqing China

**Keywords:** endothelin receptor a, immunotherapy, immunotherapy response, pan‐cancer, prognosis

## Abstract

The potential role of endothelin receptor A (EDNRA) in cancer immunotherapy has been demonstrated; however, the mechanism of its therapeutic value remains to be investigated. This study aimed to reveal the potential link between cancer immunotherapy and EDNRA in human tumours. Clinical characteristics and gene expression information were acquired from the Cancer Genome Atlas database. The correlation between EDNRA expression and immune infiltration was analysed by tumour immune estimation resource (TIMER) and tumour‐immune system interaction database (TISIDB). EDNRA expression in different cancer types were performed via qPCR. Immunohistochemistry was used to detect the relationships between EDNRA protein and immune checkpoints. The results have founded that EDNRA was differentially expressed in various tumours, and highly associated with patient's age and tumour stage. It is also of high potential prognostic value in predicting patient survival. It has been verified that the EDNRA, JAK–STAT, and TGF‐β signalling pathways are involved in cancers. In general, EDNRA positively correlated with immunomodulatory agents, immune cell infiltration, and immunotherapy markers. Immunohistochemical analysis of breast cancer tissues showed that EDNRA was positively correlated with NRP1 expression. Furthermore, patients with low EDNRA levels showed a superior response to immunotherapy. The functional study found that EDNRA expression is upregulated in MDA‐MB‐231 and HepG2 cells, and knockdown of EDNRA inhibits proliferation and migration of cells. In conclusion, the immunotherapeutic function of EDNRA was elucidated in this study. EDNRA may be an important target in tumour immunotherapy and provide new insights for tumour immunotherapy.

## 1 INTRODUCTION

Endothelin‐1, an endothelin receptor A (EDNRA), a guanine nucleotide‐binding protein, is widely expressed in humans. EDNRA encodes the endothelin‐1 (ET‐1) receptor, a peptide primarily discovered in the vascular smooth muscle that plays a role in vasoconstriction and cell proliferation.^1^, ^2^, ^3^ It has been reported that EDNRA is highly associated with many diseases, including massive atherosclerotic stroke, intracranial aneurysm, mandibular hypoplasia with alopecia,^4^, ^5^ and migraine with or without aura. Moreover, EDNRA plays an active role in various tumours. It has been verified to be related to the proliferation, invasion, migration, epithelial‐mesenchymal transition, and drug resistance of malignant tumours, including bladder cancer, papillary thyroid cancer, gastric cancer, and ovarian cancer.^3^, ^6^, ^7^ According to Laurberg et al., EDNRA is the best predictive indicator for progressed bladder cancer.^8^ According to previous reports, microRNA‐200c's downstream target gene EDNRA may demonstrably decrease the proliferation and invasion of gastric cancer cells while promoting cell death.^9^ EDNRA plays a critical role in colorectal cancer (CRC), where its upregulation is associated with tumour progression and poor patient prognosis. EDNRA regulates its own expression and that of EDN1 through the STAT3 signalling pathway, creating a positive feedback loop that promotes tumour growth and metastasis. EDNRA antagonists can inhibit the growth and migration of tumour cells.^10^ Additionally, the EDNRA/ET‐1 axis participates in angiogenesis and the reprogramming of immune cells that are linked to tumours, regulating the immune microenvironment of tumours.^1^

Massive numbers of immune cells, endothelial cells, and fibroblasts constitute the tumour microenvironment (TME).^9^, ^11^ Tumour cells evolve in a complex and highly correlated TME, including diverse immune cells, non‐tumour cells, and tumour stromal cells (endothelial cells and fibroblasts),^11^, ^12^ whereby they can communicate with their surrounding tissue. There are two primary mechanisms through which tumour cells communicate with neighbouring cells and TME elements. One involves an (ext) matrix ECM‐dependent‐dependent contact‐dependent pathway in cancer cells. The other uses soluble chemicals, including cytokines, lipid mediators, and growth regulators in a contact‐independent manner. Endothelial cells, mesenchymal cells, fibroblasts, myeloid and lymphoid inflammatory cells, and bone marrow are the primary sources of stromal cells that constitute the TME component.^11^, ^13^ Multiple cell types accumulate in the TME during various stages of tumour progression. The infiltration of immune cells, macrophages, lymphocytes, and natural killer cells is important for tumour suppression.^14^, ^15^ However, regulatory T cells (Tregs) and M2 macrophages inhibit anticancer immune responses. It is critical to study the TME in terms of the clinical impact of its composition and extent. It has been reported that favourable prognosis in patients with tumours is often accompanied by high infiltration of CD8^+^ T cells.^2^, ^11^, ^13^ Nevertheless, high infiltration of M2‐polarized macrophages is considered a marker of poor prognosis.^13^, ^16^

EDNRA expression is significantly higher in STAD patients compared to normal gastric tissue, and its high expression is associated with poor patient prognosis. ssGSEA analysis revealed a positive correlation between high EDNRA expression and the infiltration levels of macrophages and NK cells.^3^ A study has demonstrated that EDNRA plays a critical role in tumour immune suppression by modulating the secretion of EV PD‐L1, thus impacting T cell activity and reducing the efficacy of immune checkpoint blockade therapy. Treatment with macitentan to inhibit EDNRA can enhance antitumor immunity and improve the efficacy of immune checkpoint blockade therapy.^17^ Hence, there is a potential impact of EDNRA expression on pan‐cancer TME and the impact of EDNRA on cancer prognosis. Furthermore, EDNRA‐related immunomodulators and dynamic immune biomarkers were assessed to determine EDNRA's function of EDNRA in pan‐cancer and provide novel insights into the underlying mechanisms influencing TME and cancer immunotherapy.

## 2 METHODS

### 2.1 Cell lines

Human breast cancer cells (MDA‐MB‐231), liver cancer (HepG2), colon cancer (HCT116), gastric cancer (CRL‐5822), pancreatic cancer (CFPAC‐1), and lung cancer (A549) cell lines were used in this study. All cell lines were obtained from the American Type Culture Collection (ATCC) cell bank.

### 2.2 Cell Culture Conditions

The cancer cell lines were cultured in RPMI‐1640 medium (Gibco; Thermo Fisher Scientific, Inc.) supplemented with 10% FBS (Gibco; Thermo Fisher Scientific, Inc.) and 1% penicillin–streptomycin. All cells were in a cell incubator at 37°C with 5% CO_2_.

### 2.3 Data Sources

Genomic and clinicopathological data on 33 different cancer types were gathered from The Cancer Genome Atlas (TCGA) dataset and University of California Santa Cruz Xena Explorer (cohort: TCGA Pan‐Cancer). The full names and abbreviations of 33 tumours are as follows: Bladder Urothelial Carcinoma (BLCA); Cervical squamous cell carcinoma and endocervical adenocarcinoma (CESC); Cholangiocarcinoma (CHOL); Colon adenocarcinoma (COAD); Oesophageal carcinoma (ESCA); Glioblastoma multiforme (GBM); Head and Neck squamous cell carcinoma (HNSC); Kidney Chromophobe (KICH); Kidney renal clear cell carcinoma (KIRC); Kidney renal papillary cell carcinoma (KIRP); Brain Lower Grade Glioma (LGG); Liver hepatocellular carcinoma (LIHC); Lung adenocarcinoma (LUAD); Prostate adenocarcinoma (PRAD); Rectum adenocarcinoma (READ); Stomach adenocarcinoma (STAD); Thyroid carcinoma (THCA); Uterine Carcinosarcoma (UCS); Pancreatic adenocarcinoma (PAAD); Sarcoma (SARC); Acute Myeloid Leukaemia (LAML); Uveal Melanoma (UVM); Breast invasive carcinoma (BRCA); Lung squamous cell carcinoma (LUSC); Uterine Corpus Endometrial Carcinoma (UCEC); Testicular Germ Cell Tumours (TGCT); Pheochromocytoma and Paraganglioma (PCPG); Skin Cutaneous Melanoma (SKCM); Lymphoid Neoplasm Diffuse Large B‐cell Lymphoma (DLBC); Mesothelioma (MESO); Ovarian serous cystadenocarcinoma (OV); Thymoma (THYM); Adrenocortical carcinoma (ACC).

### 2.4 Expression and Prognosis Role analyse of EDNRA

The limma package in R Studio was used to analyse the differential EDNRA expression in different malignancies. We obtained EDNRA expression through comparing the expression between tumour and normal samples. The EDNRA expression exhibiting a fold change >1 after log_2_ transformation and an FDR of <0.05 were regarded as significant. The impact of EDNRA levels on several clinical indicators was examined (age and tumour phase). Additionally, using the survival package in R, univariate Cox regression analysis was performed to show the time‐dependent prognostic significance of EDNRA in 33 cancers. Survival outcomes included overall survival (OS), disease‐free survival (DFS), disease‐specific survival (DSS), and progression‐free survival (PFS).

### 2.5 Gene Set of Enrichment Analysis (GSEA)

TCGA data were used to examine the relationship between EDNRA and all genes. Pearson's correlation coefficients were obtained, and gene set enrichment assessment was performed on the genes involved in EDNRA (*p <* 0.05).

### 2.6 TIME Analyse

The immune cell infiltration value of TCGA was examined using downloads from the ImmuCellAI and TIMER2 databases (http://bioinfo.life.hust.edu.cn/web/ImmuCellAI/ and http://timer.cistrome.org/). The median() function is used to calculate the median value of the EDNRA gene expression. Patients were divided into two cohorts (low EDNRA and high EDNRA) to compare the level of immune cell infiltration within pan‐cancer based on EDNRA expression.

### 2.7 Specimens collection

The collected tissues were used for reverse transcription‐quantitative PCR (RT‐qPCR) and immunohistochemistry (IHC), which were performed as described below. All specimens collected were preserved in liquid nitrogen. Sixteen pairs of breast cancer and adjacent tissues and 12 pairs of liver cancer and adjacent tissues were used for q‐PCR detection. Thirty‐three pairs of breast cancer and adjacent tissues and 23 pairs of liver cancer and adjacent tissues were used for IHC detection.

### 2.8 IHC

Tissue samples from the First Affiliated Hospital of Chongqing Medical University (CQMU) were collected for research on breast and liver cancer. All specimens were formalin‐fixed, paraffin‐embedded, and cut into 4 μm sections, which were mounted on glass slides. For IHC, the slides were deparaffinized and rehydrated using xylene and a graded ethanol series for 30 min. Antigen retrieval was performed by microwaving the sections in sodium citrate‐hydrochloric acid buffer at 95°C for 20 min. After cooling to room temperature, endogenous peroxidase activity was blocked with 3% hydrogen peroxide. The sections were then washed three times with phosphate‐buffered saline (PBS) and blocked with normal goat serum. Primary antibodies (anti‐EDNRA (Proteintech, 19780–1‐AP) and anti‐NRP1 (Abcam, ab81321)) were applied, and the sections were incubated overnight at 4°C. Following three PBS washes, the sections were incubated with biotinylated goat anti‐mouse IgG, followed by incubation with streptavidin‐biotin‐conjugated horseradish peroxidase (HRP). After additional washing, signals were visualized using diaminobenzidine, and the sections were counterstained with haematoxylin. IHC staining intensity was scored as follows: 0, none; 1, weak; 2, medium; and 3, strong. The proportion of positive tumour cells was scored as: 0, <5%; 1, 5%–25%; 2, 26%–50%; 3, 51%–75%; and 4, >75%. The overall score was obtained by multiplying the intensity and proportion scores.

### 2.9 RNA isolation and RT‐qPCR

As previously described,^18^ we followed the manufacturer's instructions and used the TRIzol reagent to extract total RNA (Life Technologies Inc., Gaithersburg, Maryland, USA). RNA concentration was measured using a NanoDrop 2000 spectrophotometer (Thermo Scientific, Wilmington, DE, USA). Reverse transcription of 1 μg of RNA into cDNA was performed using a Reverse Transcription Kit (Promega Inc., USA). RT‐qPCR was performed on tissue pairs using an ABI 7500 real‐time PCR system (ABI, USA) with Maxima SYBR Green/ROX qPCR Master Mix (MBI Fermentas, St. Leon‐Rot, Germany) to detect ENDRA expression. Relative quantification of ENDRA mRNA expression was normalized to that of GAPDH. The primer pairs used in this study were listed in Table S1.

### 2.10 Construction of shRNA lentiviral vectors targeting ENDRA

Lentiviral vectors for ENDRA gene knockdown were constructed and packaged by Biomedicine Biotechnology. The sh1‐ENDRA group was transduced with a lentivirus carrying a shRNA sequence of TCTTCATTTAAGCCGTATATT; the sh2‐ENDRA group was transduced with a lentivirus carrying a shRNA sequence of GCTCAGGATCATTTACCAGAA; the NC group was transduced with an empty vector lentivirus; and the blank group received only fresh culture medium. The lentiviral vector for ENDRA knockdown was pLVX‐shRNA1, with a cloning site of BamHI‐EcoR1.

### 2.11 Cell Counting Kit‐8 (CCK‐8) assay

Cells from each group were collected during the logarithmic phase to prepare a single‐cell suspension, and the cell density was adjusted after counting. The cells were then seeded into 96‐well plates at a density of 1 × 10^3^ cells per well. On days 1, 2, and 3, 10 μL of CCK‐8 reagent (Beyotime Institute of Biotechnology) was added to each well. After incubating at 37°C for 1 h, the absorbance at 450 nm was measured using an xMark™ microplate spectrophotometer (BenchmarkPlus™ system, Bio‐Rad Laboratories, Inc.). Following the 3‐day period, a cell proliferation curve was generated based on the absorbance values.^19^

### 2.12 Wound Healing Assays

Logarithmic phase MDA‐MB‐231 and HepG2 cells were seeded into 6‐well plates at a density of 5 × 10^9 cells/mL, with three replicates per group. A 10 μL pipette tip was used to make uniform, smooth vertical scratches on the bottom of each well. The plates were then washed twice with PBS to remove detached cells, and images were captured under a microscope at the 0‐h time point. Subsequently, 2 mL of culture medium was added to each well, and the cells were incubated at 37°C with 5% CO_2_ for 24 h. After incubation, cell migration was observed and photographed using a microscope. The migration distance was quantified using Image J software. The migration rate was calculated using the formula: (initial scratch area − final scratch area)/initial scratch area × 100%.

### 2.13 Statistical Analysis

Statistical analyses were conducted using IBM SPSS 23.0 (Armonk, NY, USA) and GraphPad Prism 9.0 (San Diego, CA, USA). Two‐tailed Student's *t*‐tests were employed to assess statistical differences between groups. The Limma package was used to assess differentially expressed genes between tumour and normal samples. The prognostic significance of EDNRA was determined through univariate and multivariate Cox regression analyses. Correlations between gene expression levels were examined using the Spearman rank correlation test. OS and relapse‐free survival (RFS) were calculated using Kaplan–Meier curves, with differences between groups assessed by Student's *t*‐test. Statistical significance was defined as a *p <* 0.05.

### 2.14 Ethical approval

The Institutional Ethics Committee of the First Affiliated Hospital of Chongqing Medical University approved the study, which was governed by the Helsinki Declaration (2022‐k558). Written informed consent was obtained from each participant.

## 3 RESULTS

### 3.1 EDNRA Expression in Pan‐cancer

**FIGURE 1:**
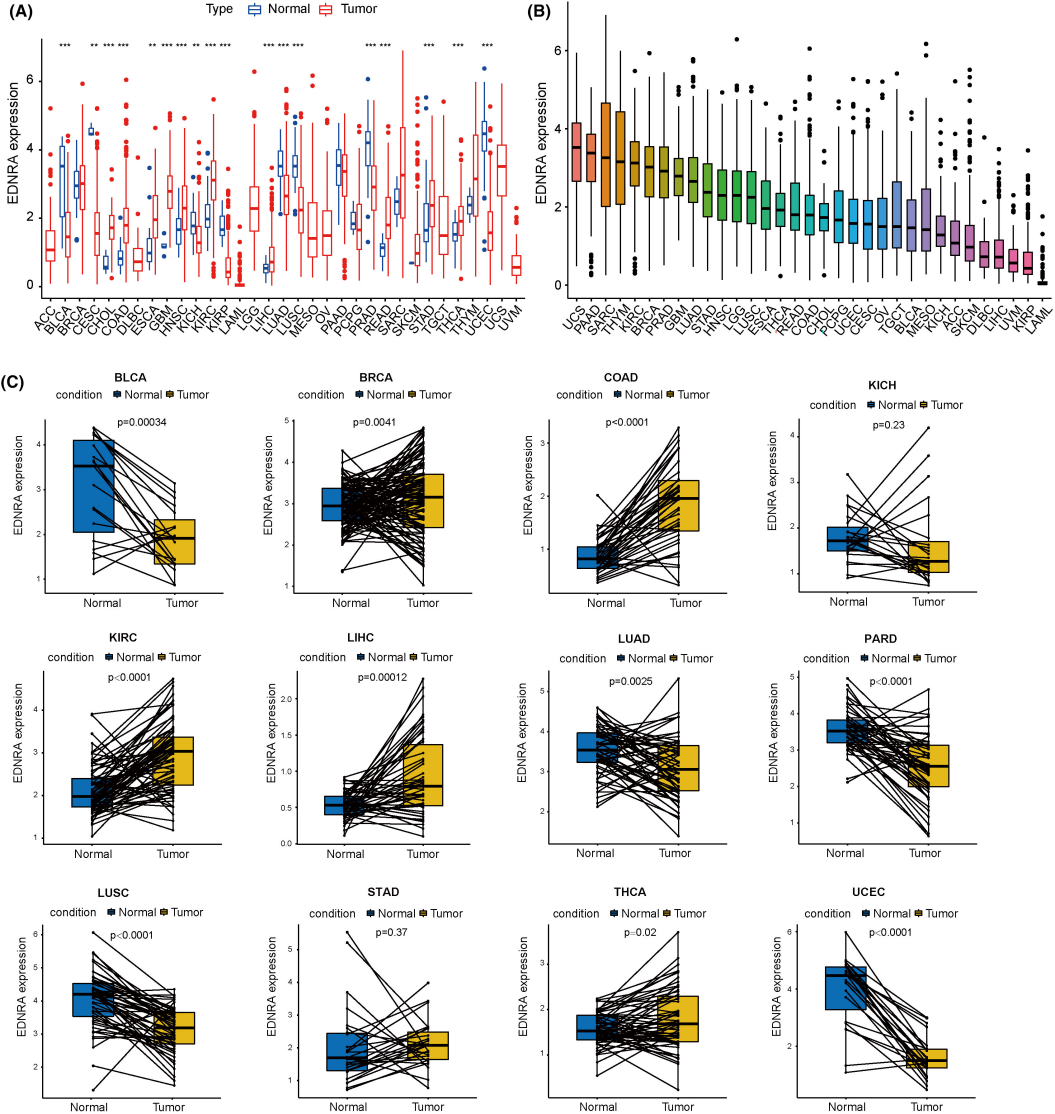
Clinical relevance of EDNRA. (A) EDNRA differential expression between tumour & normal groups in 33 malignancies and (B) EDNRA expression in 33 malignancies and (C) From the TCGA datasets of specific tumor types, pan‐cancer EDNRA differential expression between matched tumors & nearby healthy tissues (** indicates *p <* 0.01, and ***p < 0.001 between Normal and Tumour groups).

As shown in Figure 1A, differences were observed in EDNRA levels among 18 cancer types (BLCA, CESC, CHOL, COAD, ESCA, GBM, HNSC, KICH, KIRC, KIRP, LGG, LIHC, LUAD, PRAD, READ, STAD, THCA, and UCS). EDNRA showed strong expression in UCS, PAAD, and SARC, but poor expression in LAML, KIRP, and UVM (Figure 1B). Moreover, high levels of EDNRA were observed in BRCA, COAD, KIRC, LIHC, STAD, and THCA, and matched tumour and normal tissues were examined in TCGA pan‐cancer. However, low EDNRA expression was found in BLCA, KICH, LUAD, PARD, LUSC, and UCEC (Figure 1C).

**FIGURE 2:**
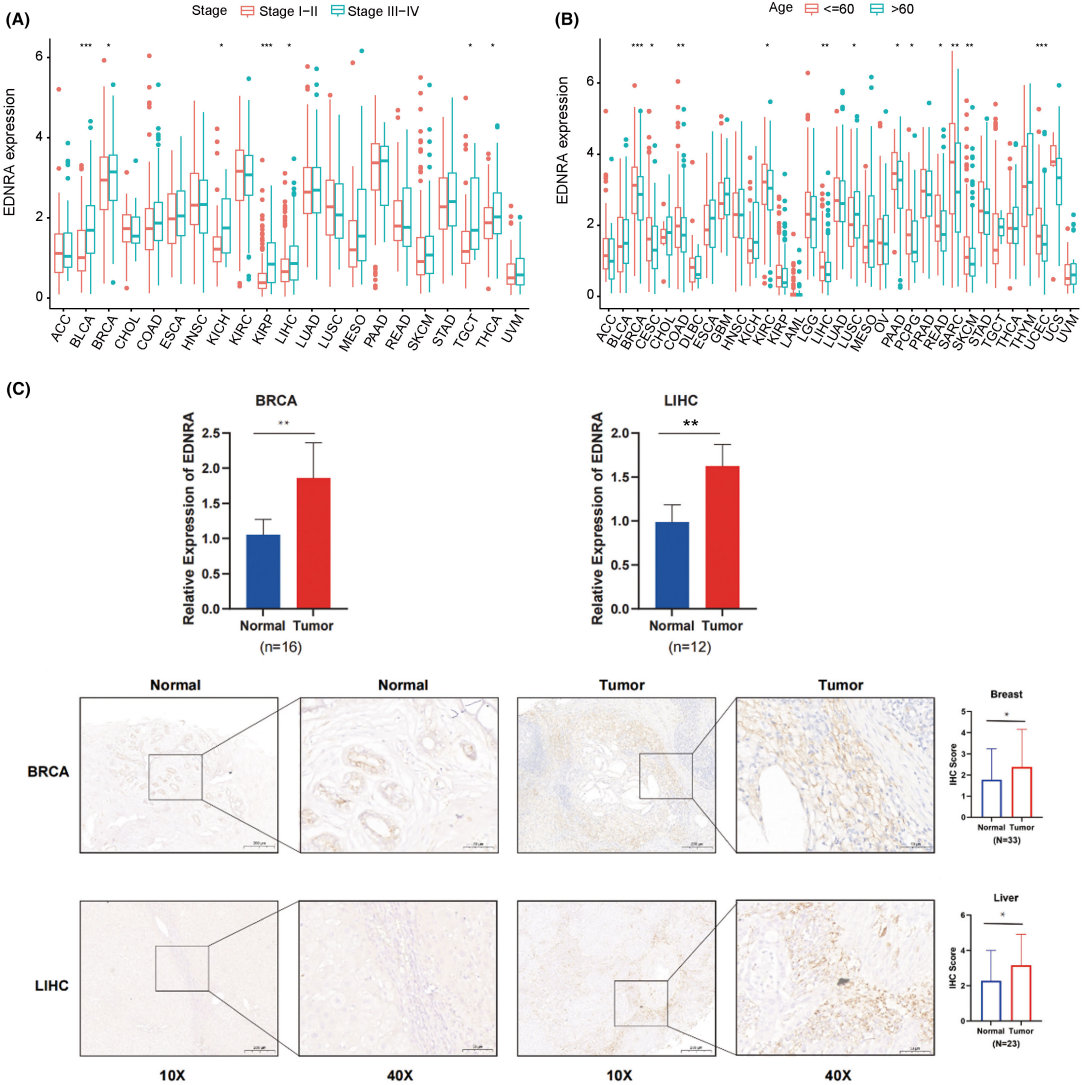
EDNRA level associated with pan‐cancer. From the TCGA datasets of specific tumour types, pan‐cancer EDNRA differential expression in 33 malignancies between (A) tumour phase (* indicates *p <* 0.05, ***p < 0.001 between Stage I‐II and Stage III‐IV) and (B) age (* indicates *p <* 0.05, **p < 0.01, ***p < 0.001 between age ≤60 and > 60 groups); (C) EDNRA differential expression in breast and liver tumours versus normal tissues. * indicates *p <* 0.05, **p < 0.01 between Normal and Tumour groups.

Furthermore, EDNRA was shown to have a strong relationship with the tumour stages of BLCA, BRCA, KICH, KIRC, LIHC, TGCT, and UVM (Figure 2A). Elderly individuals with LUSC and younger patients with BRCA, CESC, COAD, KIRC, LIHC, PAAD, PCPG, READ, SARC, SKCM, and UCS had high levels of EDNRA (Figure 2B). After the analysis of EDNRA level in the collected breast and liver cancer tissues, as expected, EDNRA was overexpressed in both cancer tissues (Figure 2C), which were consistent with our previous results.

### 3.2 Prognostic Role of EDNRA

**FIGURE 3:**
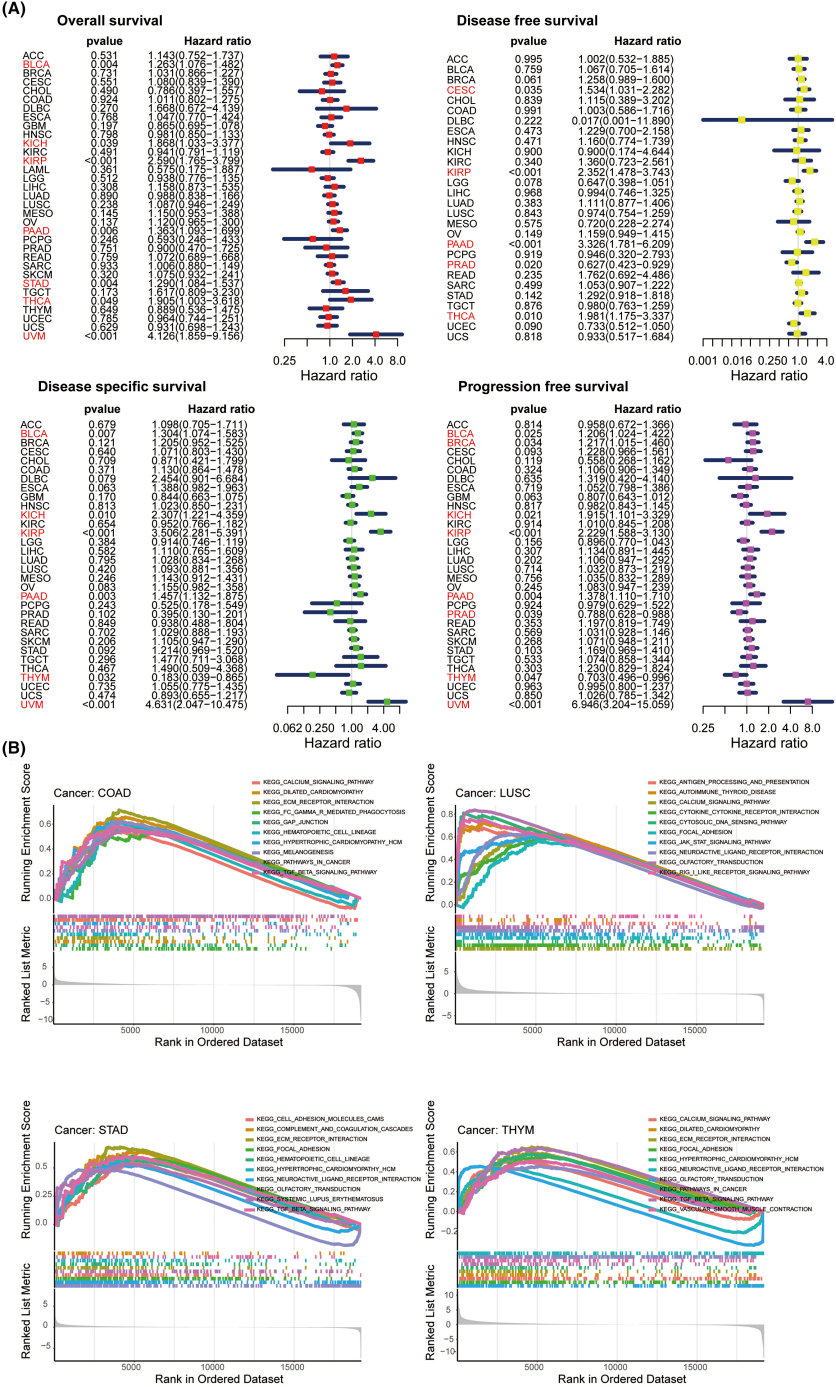
(A) Cox regression analysis using one variable using forest plots. The emphasized components suggest that ENDRA is closely linked to certain cancer kinds' prognosis. Items exhibiting hazard ratios over 1 showed a contributing factor of EDNRA to mortality; (B) KEGG database‐based GSEA results. The pathway labelled on the left and right is enriched within the groups with elevated/low EDNRA expression, respectively.

The predictive function of EDNRA in TCGA pan‐cancer was then determined using a univariate Cox regression assessment (Figure 3A). Kaplan–Meier OS results indicated that EDNRA expression was positively correlated with BLCA (HR = 1.263), KICH (HR = 1.868), KIRP (HR = 2.59), PAAD (HR = 1.363), STAD (HR = 1.29), THCA (HR = 1.905), and UVM (HR = 4.126). DFS results indicated that EDNRA was protective in patients with PRAD (HR = 0.627) and risk in individuals with CESC (HR = 1.534), KIRP (HR = 2.352), PAAD (HR = 3.326), and THCA (HR = 1.981). Furthermore, as DSS forest plot illustrated, EDNRA expression was negatively correlated with DSS in THYM (HR = 0.183) patients and positively correlated in BLCA (HR = 1.304), KICH (HR = 2.307), KIRP (HR = 3.506), PAAD (HR = 1.457), and UVM (HR = 4.631) patients. PFS results indicated a protective factor of EDNRA in PRAD (HR = 0.788) and THYM (HR = 0.703) patients and a risk factor for BLCA (HR = 1.206), BRCA (HR = 1.217), KICH (HR = 1.915), KIRP (HR = 2.229), PAAD (HR = 1.378), and UVM (HR = 6.946).

### 3.3 GSEA of EDNRA

Potential pathways with EDNRA involved in TCGA pan‐cancer were predicted using Kyoto Encyclopedia of Genes and Genomes (KEGG) pathway database‐based GSEA. EDNRA has been shown to be strongly connected to immune‐related pathways, including the JAK–STAT and TGF‐β signalling pathways. Therefore, EDNRA was identified as an imperative gene in cancers (Figure 3B).

### 3.4 Underlying Association of Immune Cell Infiltration and EDNRA

**FIGURE 4:**
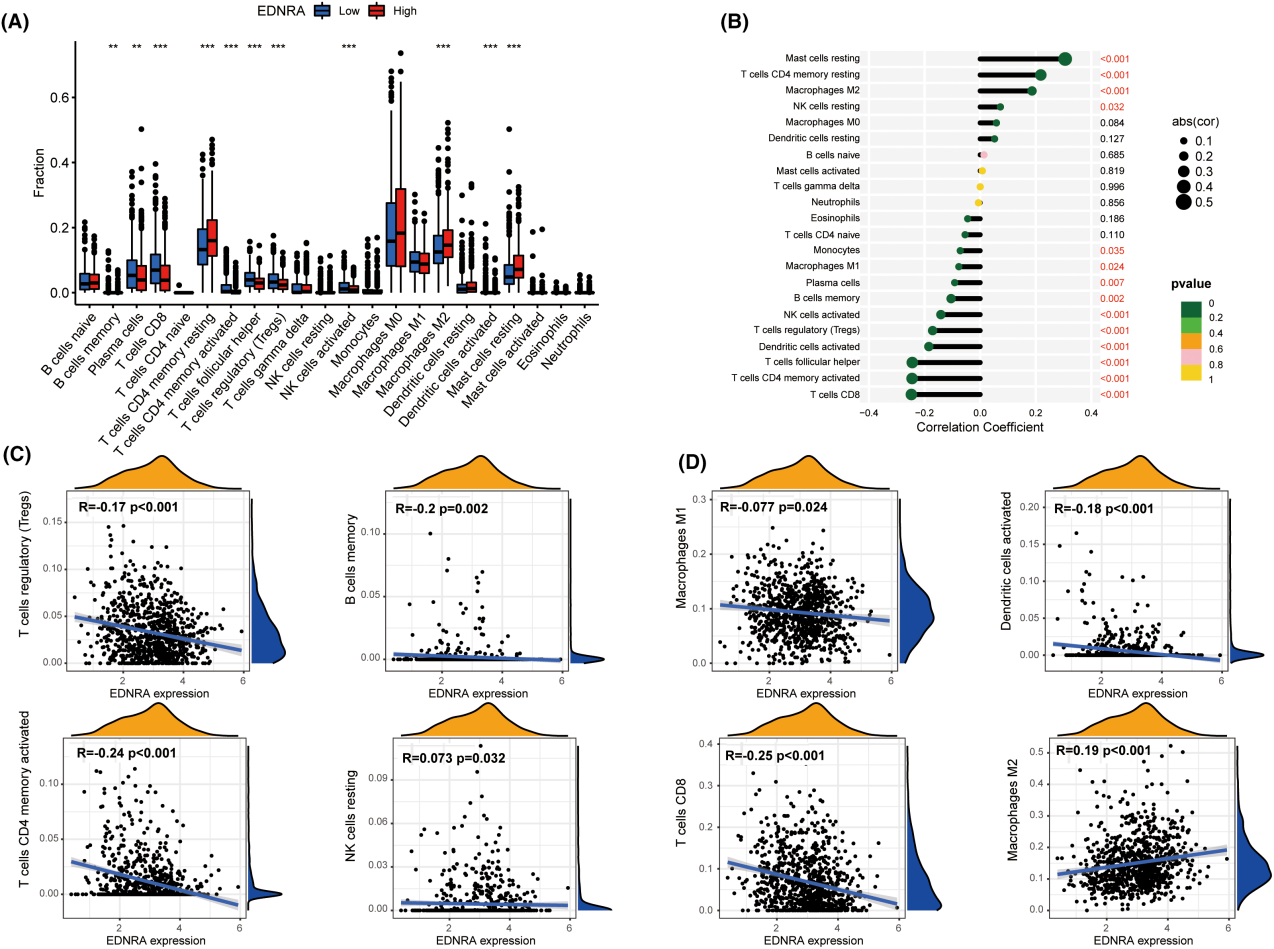
Analysis of immune cell infiltration. (A–D) Association of EDNRA with immune cell infiltration degree. **p < 0.01 and ***p < 0.001.

A highly expressed differential EDNRA was found in B lymphocytes, CD8^+^ T lymphocytes, CD4^+^ T lymphocytes, NK lymphocytes, macrophages, dendritic cells, and mast cells. EDNRA was evidently associated with TCGA pan‐cancer, including mast cells, CD4^+^ T cells, NK cells, macrophages, monocytes, dendritic cells, plasma cells, and CD8^+^ T cells (Figure 4A,B). Furthermore, ENDRA positively correlated with mast cell infiltration (*p <* 0.001), memory CD4^+^ T cell infiltration (*p <* 0.001), M2 macrophage infiltration (*p <* 0.001), and NK cell infiltration (*p* = 0.032). It was also associated with monocyte infiltration (*p* = 0.035), M1 macrophage infiltration (*p* = 0.024), plasma cell infiltration (*p* = 0.007), memory B cell infiltration (*p* = 0.002), activated NK cell infiltration (*p <* 0.001), activated dendritic cell infiltration (*p <* 0.001), activated memory CD4^+^ T cell infiltration (*p <* 0.001), and CD8^+^ T cell infiltration (*p <* 0.001) (Figure 4B–D).

### 3.5 EDNRA and Immune‐Related Factors in Relation

**FIGURE 5:**
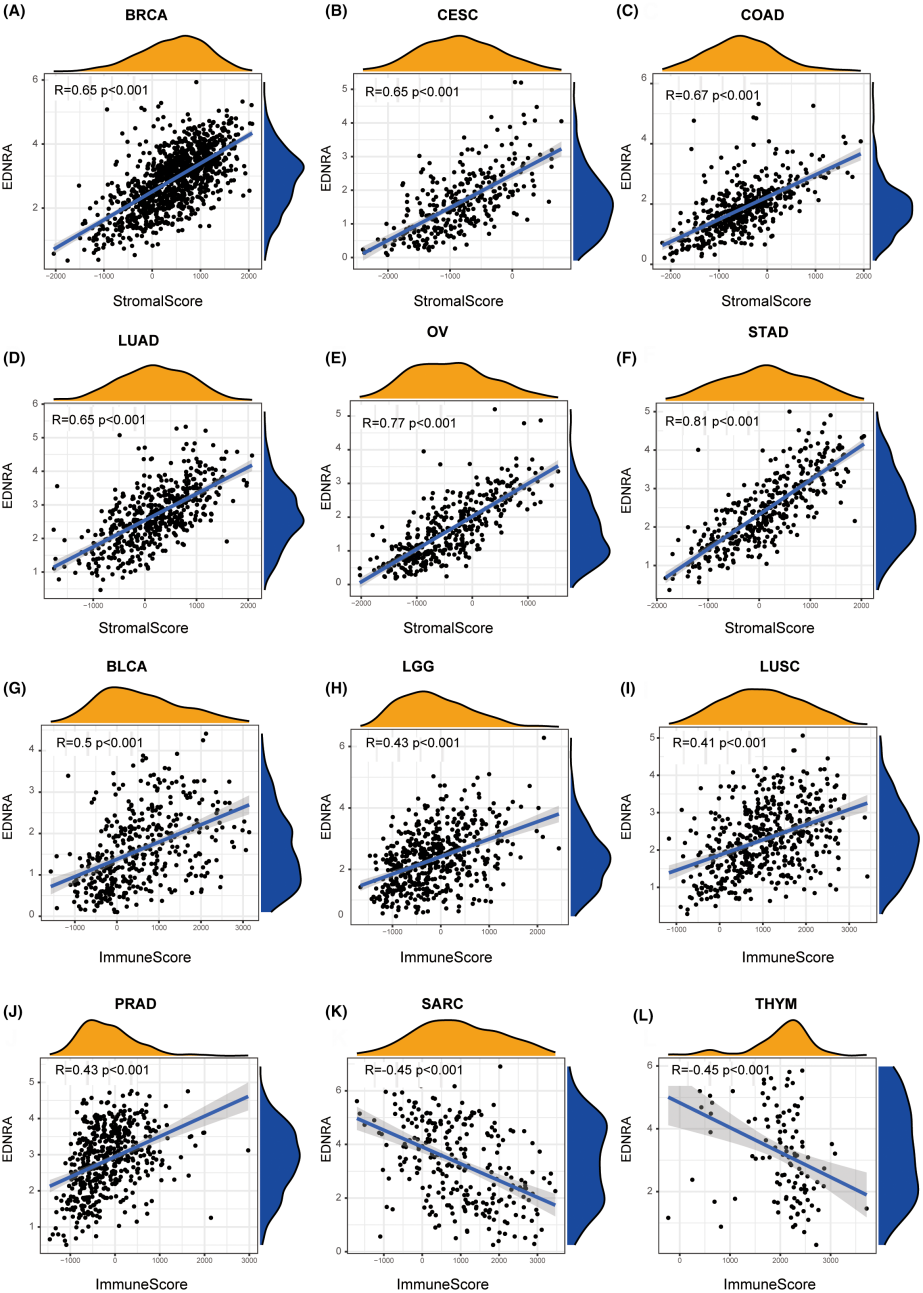
Immune cell infiltration as well as EDNRA and ESTIMATE scores correlation. (A–L) Estimated scores include the tumour purity, immunological score, and stromal value (the presence of stromal cells within the tumour tissue). The CIBERSORT method calculates immune cell infiltration. R more than 0.5 and *p <* 0.05 indicate the presence of correlation.

**FIGURE 6:**
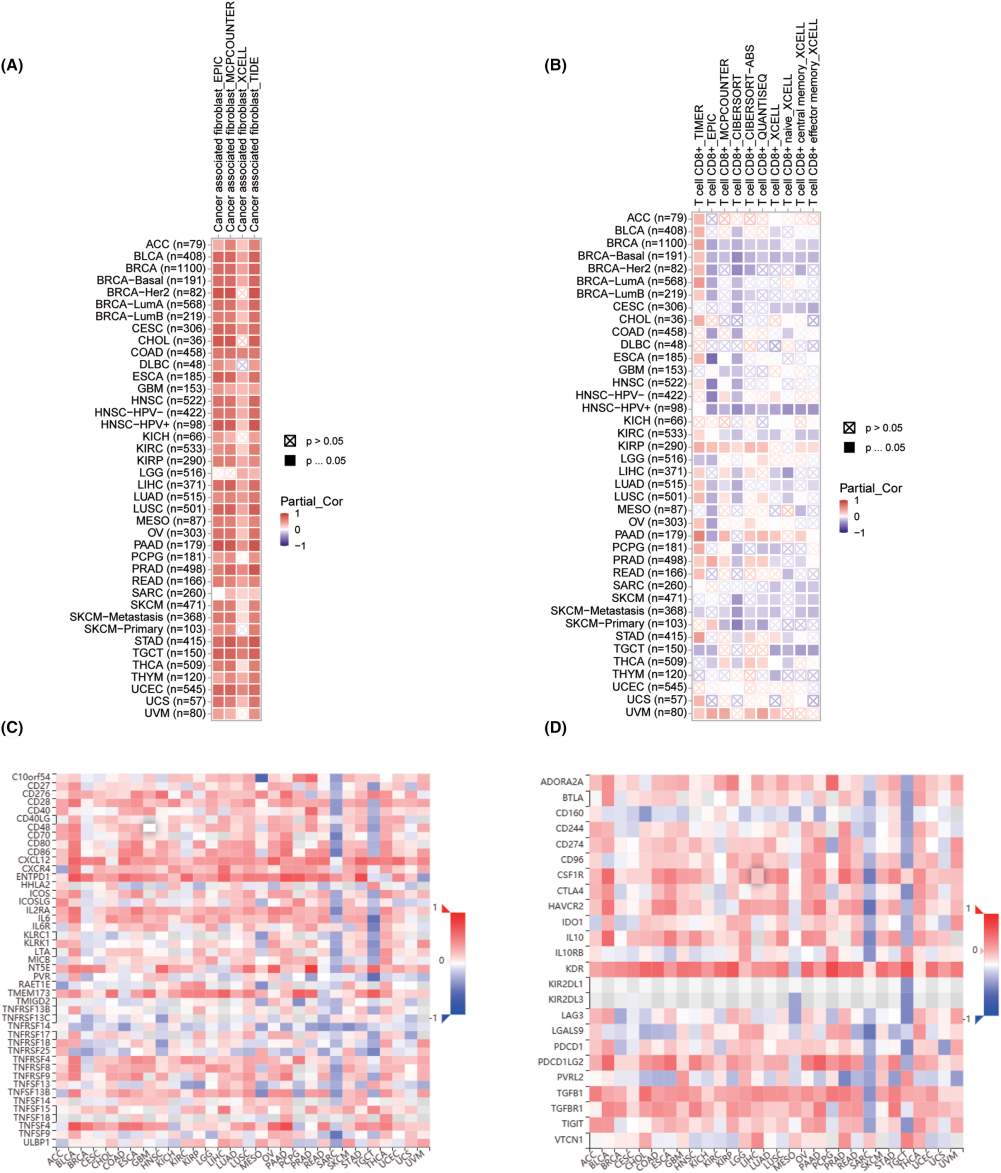
Relationship between EDNRA and tumour microenvironment. (Correlations involving EDNRA & CD8 + T cell infiltrating levels shown by the TIMER2 database in (A), CAF cell infiltrating levels shown by the TIMER2 database in (B), immunosuppressant levels shown by the TIMER2 database in (C), and immune activator levels shown by the TIMER2 database in (D). *p < 0.05, **p < 0.01, ***p < 0.001, and ****p < 0.0001.)

**FIGURE 7:**
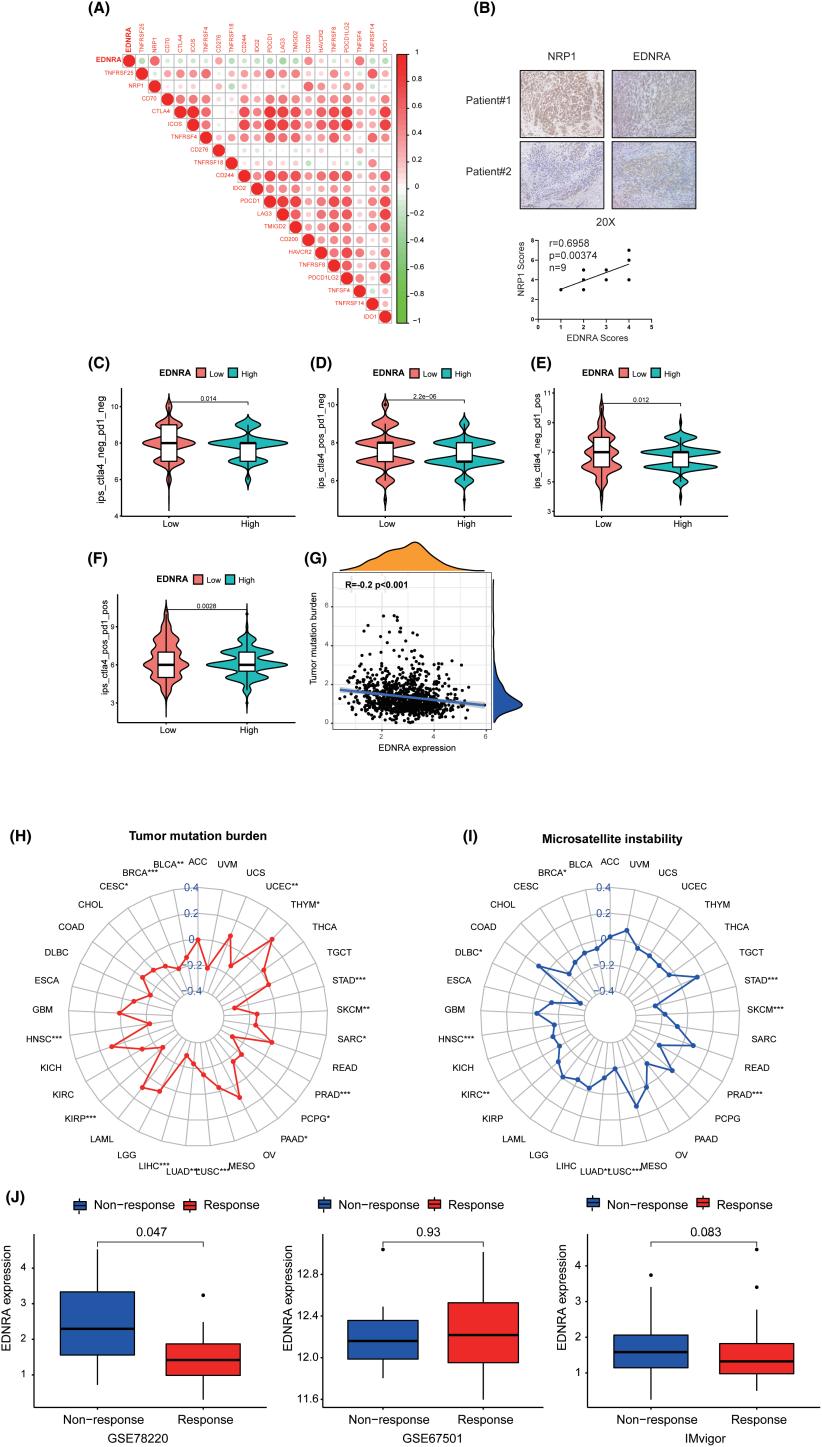
Relationship between EDNRA and immune checkpoint inhibitors. (A) Association of EDNRA with immune checkpoint inhibitors. (B) Relationship between NRP1 and ENDRA in breast cancer tissues. (C–F) Association of immunosuppressant reactivity in pan‐cancer patients. (G) Tumour mutation burden (TMB) of EDNRA; (H‐J) Association of EDNRA with TMB, MIS, and response of immunotherapy. *p < 0.05, **p < 0.01, and ***p < 0.001.

EDNRA stromal and immunological scores are shown in Figure 5. Notably, EDNRA demonstrated a positive correlation with BRCA, CESC, COAD, LUAD, OV, and STAD stromal scores (Figure 5A–F). It was positively correlated with BLCA, LGG, LUSC, and PRAD immune scores but negatively correlated with SARC and THYM immune scores (Figure 5G–L). It was also positively correlated with cancer‐associated fibroblasts (CAFS) (Figure 6A,B). The correlation analysis of 24 immunosuppressants indicated that EDNRA was positively correlated with ADORA2A, CSF1R, KDR, TGFB1, and TGFBR1 in cancers, including BLCA, CHOL, ESCA, READ, and STAD (Figure 6C). The effects of the 45 immunostimulants on EDNRA were analysed. EDNRA positively correlated with ENTPD1, TMEM173, and TNFSF4 in BLCA and PAAD (Figure 6D). The transcript levels of immune checkpoint genes, including NRP1, CD276, CD200, and TNFSF4, were also notably associated with EDNRA. Furthermore, a negative correlation was observed between EDNRA and TNFRSF25, TNFRSF18, IDO2, LAG3, and IDO1 (Figure 7A). IHC analysis of breast cancer tissues showed that EDNRA positively correlated with NRP1 (Figure 7B). The immune epigenetic score was used to assess whether risk scores could predict patient responses to immune checkpoint inhibitors (ICI). The immune cell proportion score (IPS) was markedly higher in the low‐risk group, indicating a superior chance of ICI application in low‐risk patients (Figure 7C–F). Our data showed that EDNRA was inversely associated with Tumour Mutational Burden (TMB) in pan‐cancer analysis (*r* = −0.2, *p <* 0.001) (Figure 7G). EDNRA was suggested to be positively correlated with UCEC, THYM, and LGG. However, negative relationships were found for BLCA, BRCA, CESC, HNSC, KIRP, LIHC, LUAD, LUSC, PAAD, PCPG, SARC, SKCM, and STAD. BRCA, DLBC, HNSC, LUAD, LUSC, PRAD, SKCM, and STAD all demonstrated negative relationships with microsatellite instability (MIS) (Figure 7H,I).

### 3.6 Immunotherapeutic Response of EDNRA

In the GSE78220 cohort, patients with low EDNRA levels tended to respond to immunotherapy, whereas the other two cohorts showed no significant differences (Figure 7J). Hence, patients with low EDNRA levels show a superior response to immunotherapy.

### 3.7 EDNRA Interference Suppresses Proliferation and Migration in MDA‐MB‐231 and HepG2 Cells

**FIGURE 8:**
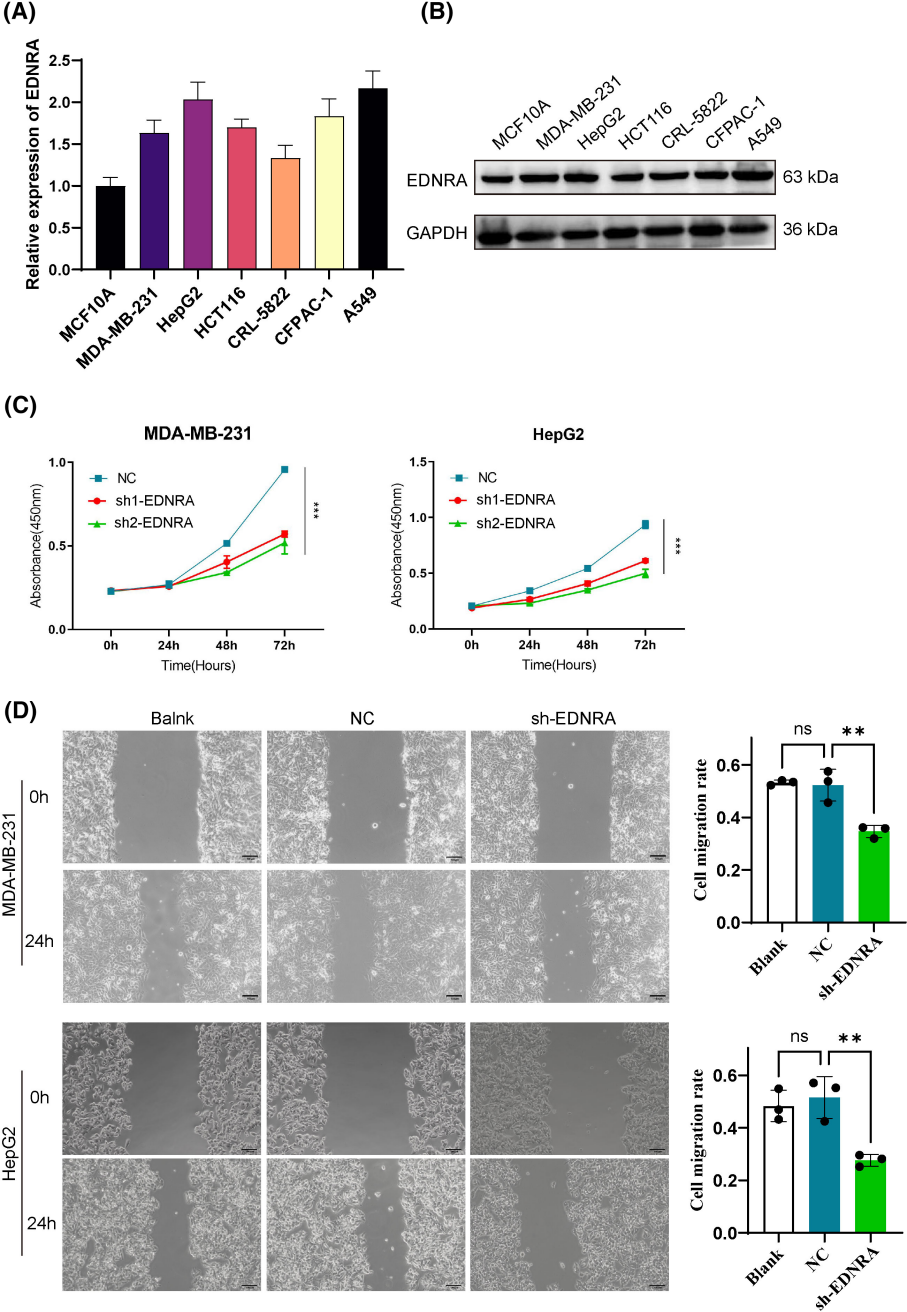
EDNRA expression was detected in cancer cells. (A) EDNRA mRNA was detected in six cancer lines (MDA‐MB‐231, HepG2, HCT116, CRL‐5822, CFPAC‐1, and A549); EDNRA interference suppress (B) cell viability and (C) cell migration in MDA‐MB‐231 and HepG2 cells. ** indicates *p <* 0.01; *** indicates *p <* 0.001 compared with NC.

To validate the bioinformatics results for EDNRA, we utilized six tumour cell lines and assessed the gene expression of EDNRA through qPCR. Compared to MCF10A, EDNRA expression was elevated in breast cancer cells (MDA‐MB‐231). Notably, EDNRA gene levels were higher in MDA‐MB‐231, HepG2, CFPAC‐1, and A549 cells (Figure 8A). Additionally, as shown in Figure 2, EDNRA is overexpressed in both breast cancer and liver cancer tissues. To further investigate the role of EDNRA in breast and liver cancers, we designed shEDNRA to knock down EDNRA expression in MDA‐MB‐231 and HepG2 cell lines. As anticipated, shEDNRA treatment significantly inhibited the proliferation and migration of MDA‐MB‐231 and HepG2 cells (Figure 8B,C).

## 4 DISSCUSION

An integrated study of EDNRA expression differences in normal and tumour tissues demonstrated the potential of EDNRA as an immunotherapeutic agent for a variety of tumour types. As a G protein‐coupled receptor for endothelin, EDNRA is expressed in vascular smooth muscle cells, the heart, kidney, and neuronal cells.^20^ As previously reported, the ET‐1/EDNRA axis can promote cell growth, differentiation, and invasiveness in various tumours. Furthermore, EDNRA overexpression is associated with the occurrence and progression.^3^, ^4^ Numerous studies have indicated the imperative role of EDNRA in tumour immunity. Jin et al. demonstrated that a new prognostic signature can greatly stratify patients with muscle‐invasive bladder cancer into various risk groups, providing a basis for intensive therapy in high‐risk individuals with poor survival outcomes.^21^ Hong et al. discovered the importance of 21 immune‐related key genes (including EDNRA) in the HCC microenvironment, further revealing the potential molecular mechanisms of HCC occurrence and progression.^22^ Hence, the potential mechanism of EDNRA in the TME was explored via TCGA pan‐cancer approach, hoping to discover new targets for immunotherapy of different tumours.

First, EDNRA's prognostic function of EDNRA in the TCGA pan‐cancer was established. Multiple cancers, including BLCA, CESC, CHOL, COAD, ESCA, GBM, HNSC, KICH, KIRC, KIRP, LGG, LIHC, LUAD, PRAD, READ, STAD, THCA, and UCS were differentially expressed. High EDNRA levels were detected in BRCA, COAD, KIRC, LIHC, STAD, and THCA, whereas low levels were detected in BLCA, KICH, LUAD, PARD, LUSC, and UCEC. Furthermore, EDNRA might also be related to patient age, and tumour classification for EDNRA is associated with clinicopathological features. This survival analysis revealed a risk factor for EDNRA in most tumours, and EDNRA had the highest HR in KIRP and UVM. Nevertheless, EDNRA studies on KIRP and UVM are rare and can provide new research ideas for other researchers.

As killer cells of the T lymphocyte population,^23^ cytotoxic CD8^+^ T cells contribute to cellular immunity, especially in tumours. Cytotoxic CD8^+^ T cells are actively activated and remembered by CD4^+^ T cells.^24^, ^25^ As verified using the TIMER2 database, EDNRA was inversely correlated with NK, CD4^+^, and CD8^+^ T cells. Thus, the inhibitory effect of EDNRA on most tumour types was verified. Tregs exert an inhibitory effect, helping malignant tumour cells escape cytotoxic CD8^+^ T cells attacks.^26^ Treg infiltration was also negatively associated with EDNRA, suggesting its limited function, despite the abundance of cytotoxic CD8^+^ T cells. Additionally, negative and positive correlations were found between EDNRA and M1‐ and M2‐like macrophages, respectively, suggesting a direct or indirect effect of EDNRA on macrophage polarisation.

TME stromal cells and tumour‐adaptive immune cells may expedite tumour development. Immune escape may result from immune cell reconfiguration in tumours.^27^ As indicated by GSEA, EDNRA is involved in pathways associated with immunological control. Furthermore, positive correlation between EDNRA and CAF was indicated by TIMER2 database. Our findings indicate that EDNRA directly or indirectly modulates CAF infiltration. Regarding the above results, a discussion was held regarding how ENDRA affected immunological checkpoints, immunosuppressive genes, chemokines, and chemokine receptors. EDNRA was favourably correlated with immune checkpoints and immunosuppressive genes, such as NRP1, CD276, CD200, and TNFSF4. This suggests that EDNRA is closely related to immune regulation, and that tumour patients with high EDNRA levels might exhibit an immunosuppressive state.

Furthermore, two immunotherapy biomarkers, TMB and MSI, indicate an intimate association with EDNRA in certain cancers. Typically, when there are increasing somatic mutations generated in a tumour, more neoantigens are likely to be generated, and TMB provides a meaningful estimate of the tumour neoantigen burden.^28^ Conversely, MSI, a severely mutated phenotype caused by a defect in DNA mismatch repair, may serve as a predictor for immunotherapy.^28^, ^29^ In BRCA, LUAD, and LUSC, EDNRA showed a negative connection with TMB and MSI. but a positive connection with two biomarkers in THYM and UCEC, implying a possible indirect consequence of EDNRA on immunotherapy response in these cancers. Subsequently, the influence of EDNRA on the response to immunotherapy was investigated. Substantial differences were observed in the GSE78220 dataset. Therefore, EDNRA may affect pan‐cancer immunotherapy response.

Taken together, EDNRA was comprehensively evaluated, and its potential role as a prognostic indicator and its immunomodulatory effects in patients were identified. In summary, as a possible immune‐related gene, EDNRA can be used as a target for tumour immunotherapy.

## AUTHOR CONTRIBUTIONS

**Mengxue Wang:** Conceptualization (equal); writing – original draft (equal). **Long Wang:** Data curation (equal); visualization (equal). **Xunjia Li:** Data curation (equal); visualization (equal). **Meng Dai:** Data curation (equal); visualization (equal). **Bo Sheng:** Conceptualization (equal); writing – original draft (equal); writing – review and editing (lead).

## FUNDING INFORMATION

This study was funded by the Project of Chongqing Education Commission Science and Technology Foundation (CSTB2023NSCQ‐BHX0090).

## CONFLICT OF INTEREST STATEMENT

It was claimed that there were no financial or commercial ties that may have created a conflict of interest in the study's implementation.

## Supporting information


Table S1.

## Data Availability

Publicly available datasets were analysed, which is located here: GSE78220 and GSE 67501 from the GEO database (https://www.ncbi.nlm.nih.gov/geo/), 33 different types of cancer from the TCGA database, and the IMvigor210 cohort from a published research.
