# Supplementary material for: EDNRA regulates the tumour immune environment and predicts the efficacy and prognosis of cancer immunotherapy
Source: J Cell Mol Med. 2024 Nov 27;28(22):e70172. doi: 10.1111/jcmm.70172 (PMC11600291; doi:10.1111/jcmm.70172)
Supplement: Supplementary file 1 — Table S1. [file JCMM-28-e70172-s001.docx]

Supplementary Table 1. Primer sequences

| Primers | Primer sequence (5ˊ-3΄) |
| --- | --- |
| EDNRA-F | CCTCAACCTCTGCGCTCTTA |
| EDNRA-R | CGAAGAGCCACCAGTCCTTT |
| GAPDH-F | GGAGCGAGATCCCTCCAAAAT |
| GAPDH-R | GGCTGTTGTCATACTTCTCATGG |
